# Thick filament‐associated myosin undergoes frequent replacement at the tip of the thick filament

**DOI:** 10.1002/2211-5463.13379

**Published:** 2022-02-20

**Authors:** Emi Ichimura, Koichi Ojima, Susumu Muroya, Ken Kobayashi, Takanori Nishimura

**Affiliations:** ^1^ 12810 Research Faculty of Agriculture Graduate School of Agriculture Hokkaido University Sapporo Japan; ^2^ Muscle Biology Research Unit Division of Animal Products Research Institute of Livestock and Grassland Science, NARO Tsukuba Japan

**Keywords:** myofibril, myosin, skeletal muscle, thick filament

## Abstract

Myosin plays a fundamental role in muscle contraction. Approximately 300 myosins form a bipolar thick filament, in which myosin is continuously replaced by protein turnover. However, it is unclear how rapidly this process occurs and whether the myosin exchange rate differs depending on the region of the thick filament. To answer this question, we first measured myosin release and insertion rates over a short period and monitored myotubes expressing a photoconvertible fluorescence protein‐tagged myosin, which enabled us to monitor myosin release and insertion simultaneously. About 20% of myosins were replaced within 10 min, while 70% of myosins were exchanged over 10 h with symmetrical and biphasic alteration of myosin release and insertion rates. Next, a fluorescence pulse‐chase assay was conducted to investigate whether myosin is incorporated into specific regions in the thick filament. Newly synthesized myosin was located at the tip of the thick filament rather than the center in the first 7 min of pulse‐chase labeling and was observed in the remainder of the thick filament by 30 min. These results suggest that the myosin replacement rate differs depending on the regions of the thick filament. We concluded that myosin release and insertion occur concurrently and that myosin is more frequently exchanged at the tip of the thick filament.

## Graphical abstract

Approximately 300 myosins form a bipolar thick filament in skeletal muscle cells where myosin insertion and release coincide. Our live imaging and pulse‐chase assays demonstrated that myosin is more frequently exchanged at the tip of the thick filament than the rest, indicating that the myosin replacement rate differs depending on the region of the thick filament.

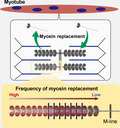

**Abbreviations**
ACD: assembly competence domain
DM: differentiation medium
FRAP: fluorescence recovery after photoconversion
GM: growth medium
KikGR: Kikume Green‐Red
LMM: light mero myosin
Mf: mobile fraction
MybpC: myosin binding protein C
Myh: myosin heavy chain
SE: standard error
t_1/2_
: half‐life

Skeletal muscle tissue consists of a bundle of myofibers filled with myofibrils, a specific organelle for muscle contraction. Myofibrils are composed of the regularly repeating minimum contraction unit, the sarcomere, which in turn is made up of more than 20 types of myofibrillar proteins. Muscle contraction is induced by the interdigitation between the thin filament and the thick filament in the sarcomere [1, 2, 3]. The main myofibrillar proteins of the thin filament are sarcomeric‐α‐actin, tropomyosin, troponin complex, and nebulin [4]. The thin filament spans from the Z‐band toward the M‐lines with nebulin [5, 6]. The thick filament contains myosin and myosin‐associated proteins, such as myosin‐binding protein C (MybpC), myomesin, and connectin/titin. MybpC bundles polymerized myosin at multiple sites, and myomesin ties the thick filament together at the M‐lines [7, 8, 9]. A single polypeptide of a giant protein, connectin/titin, extends from the Z bands to the M‐lines and interacts with MybpC and myosin on the thick filaments to maintain the thick filament at the center of the sarcomere [5, 10, 11, 12]. Thus, the sarcomere structure is made up of extensively organized myofibrillar protein complexes.

Myosin, one of the most abundant myofibrillar proteins, is a significant component of the thick filament. The myosin molecule is a hexamer composed of two myosin heavy chains (Myh), two essential light chains, and two regular light chains. The head region of myosin functions as ATPase when it interacts with actin in the thin filament. The rod region of myosin consists of the α‐helical coiled‐coil S2 domain and light meromyosin (LMM), the latter of which is responsible for myosin polymerization and incorporation into the thick filament [13]. In the LMM, the assembly competence domain (ACD), 29 amino acid residues that are located near the C‐terminal region of sarcomeric Myhs and conserved among species, is necessary but not sufficient to form the thick filament [14]. A cluster of four positive amino acid residues located in the C‐terminal region of the ACD is crucial for *in vitro* myosin filament assembly in test tubes and incorporation into the thick filament in cultured cardiac cells [15]. *In vitro*, biochemical studies have shown that the length of the myosin filament varies between 0.2 and over 5 µm according to the pH and ionic strength of the polymerization buffer [16, 17]. In contrast, *in vivo* skeletal muscle cells spontaneously create a bipolar‐shaped thick filament of 1.6 µm in length [18]. Thus, muscle cells have an instinctive ability to generate an authentic thick filament. However, the mechanism by which approximately 300 myosins are assembled into the thick filament in the skeletal muscle cells is still not fully understood.

The protein turnover process occurs within all living organisms. In the myofibrils of skeletal muscle cells, damaged or unnecessary proteins are replaced by new ones. Although myofibrils are highly organized protein complexes, each myofibrillar protein in the sarcomere component must be exchanged with another. We previously used a live imaging technique to study myosin replacement in the thick filament of cultured myotubes and found that eGFP‐tagged myosin (eGFP‐Myh3) was exchanged in myofibrils within 10 h [19]. However, it remains unclear (a) how rapidly myosin is replaced in the thick filament and (b) whether myosin is uniformly replaced within the thick filament. In the present study, we attempted to address these questions. First, we monitored the myosin replacement in the myofibrils over a short interval. Next, we determined the pattern of myosin insertion and release in the thick filament using a fluorescence pulse‐chase assay. Our findings showed that thick filament‐associated myosin molecules were exchanged within 7 min and that the frequency of myosin replacement varied depending on the region of the thick filament.

## Methods

### Experimental animals

All experiments were performed using primary muscle cells from chick embryos. Experimental animals were reared as outlined in the Hokkaido University and NARO guidelines for the care and use of laboratory animals. The relevant committees of Hokkaido University and NARO approved this study.

### Cell culture and transfection

Skeletal muscle cells were obtained from day 11 chick embryo pectoral muscles as previously described [19]. In brief, connective tissues were removed from muscles under a stereomicroscope. Minced muscle was incubated in 0.025% trypsin solution (Thermo Fisher Scientific, Tokyo, Japan) for 25 min at 37°C. To stop trypsinization, growth medium [GM: 10% (v/v) horse serum (Thermo Fisher Scientific), 10% (v/v) embryo extract, and 1% (v/v) penicillin–streptomycin–glutamine ×100 (Thermo Fisher Scientific) in minimum essential medium (Thermo Fisher Scientific)] was added to the cell suspension. After centrifugation at 600 ***g*** for 5 min, the precipitated cell fraction was resuspended in GM and then filtered through 100 and 40 µm cell strainers to eliminate cellular debris (CORNING, Tokyo, Japan). Cells were cultured on noncoating dishes for 50 min in an incubator to remove contaminated nonmuscle cells. Nonadherent cells were collected and seeded at 10 000 cells per cm^−2^ on glass‐bottom dishes (Iwaki, Shizuoka, Japan) for live‐imaging assays or on Lab‐Tek chamber slides (Thermo Fisher Scientific) for fluorescence pulse‐chase assays; both the glass‐bottom dishes and chamber slides were coated with poly‐l‐lysine (Sigma‐Aldrich, Tokyo, Japan) and collagen Type Ⅰ‐A (Nitta Gelatin, Osaka, Japan). The medium was shifted from GM to differentiation medium [DM: 11% (v/v) horse serum, 3% (v/v) embryo extract, and 1% (v/v) penicillin–streptomycin–glutamine ×100 in minimum essential medium] on the day of transfection or on the day after seeding. Cells were transfected with expression vectors using Lipofectamine®︎ LTX and Plus reagents (Thermo Fisher Scientific). DM was changed every two days.

### cDNA constructs

Mouse cDNA for *Myh3* (45–5868 in NM_001099635) was cloned into the HaloTag®︎ vector (Promega KK, Tokyo, Japan) or a humanized monomeric Kikume green‐red 1 vector (KikGR1; Medical and Biological Laboratories, Nagoya, Japan) [19]. Tags were located at the N‐terminal region of Myh3: Halo‐Myh3 and KikGR‐Myh3 were expressed in cells. The inserted sequences of all constructs were verified with a 3730 DNA Analyzer (Applied Biosystems, Tokyo, Japan).

### Fluorescence recovery after photoconversion (FRAP) assay

FRAP assay was performed with a Leica TCS SP5 confocal microscope (Leica Microsystem, Tokyo, Japan) equipped with a microscope incubation system (Tokai Hit, Shizuoka, Japan) to observe live cells [19]. A part of myofibrils was irradiated with UV light at 15% power of maximum output for 8 s to convert KikGR fluorescence green to red. Images were obtained every 10 min after photoconversion. The green and red fluorescence of KikGR were detected at excitation wavelengths of 3% 488 nm and 15% 543 nm with 500–540 nm and 600–700 nm band‐pass filters. The identical focal plane was selected for the quantification of fluorescence intensity. The green fluorescence intensity of KikGR‐Myh3 in the photoconverted area was normalized with that of the nonconverted area to correct for the influence of photobleaching. We adjusted the red intensity of KikGR‐Myh3 using a correction value. This value was derived from the slope of the red fluorescence intensity of KikGR‐Myh3 from 500 min to 600 min with a linear change. The red fluorescence was normalized by adding the value of multiplying the correction value and the elapsed time. The relative green fluorescence intensity at pre‐photoconversion was defined as 1, and the relative green fluorescence intensity just after photoconversion was defined as 0. The relative red fluorescence intensity just after photoconversion was defined as 1, and the relative red fluorescence intensity just after photoconversion in the non‐photoconverted area was defined as 0. Normalized fluorescence data were applied to recovery curve‐fitting using the following exponential formula by imagej 1.52a software (National Institutes of Health, Bethesda, MD, USA).

$$FI=Mf*(1‐e^{‐b*t})+c$$

FI is the normalized fluorescence intensity, and t is the elapsed time after bleaching. The mobile fraction (Mf) was the maximum change value of fluorescence intensity. The half‐life (t_1/2_) was calculated as the time required for the fluorescence intensity change to reach half of Mf. Mf and t_1/2_ were used as indicators of fluorescence recovery (green fluorescence) and fluorescence reduction (red fluorescence) rates of KikGR‐Myh3.

### Fluorescence pulse‐chase assay

Day 6 myotubes expressing Halo‐Myh3 were labeled with Oregon‐Green®︎ ligand (green fluorescence; Promega) at a final concentration of 0.3% in DM for 16 h. Following washout, myotubes were reacted with TMR ligand (red fluorescence, Promega) at a final concentration of 0.1% in minimum essential medium (Thermo Fisher Scientific) to label newly synthesized Halo‐Myh3 for 7, 15, and 30 min. Myotubes were fixed with 4% paraformaldehyde in PBS (Nacalai Tesque, Kyoto, Japan), rinsed with 0.5% Triton X‐100 in PBS for 5 min three times, and mounted with media containing 4’,6‐diamidino‐2‐phenylindole (DAPI; Vector Laboratories, Burlingame, CA, USA). Samples were analyzed using an LSM 700 Confocal Laser Scanning Microscope (Carl Zeiss, Tokyo, Japan) equipped with a Plan‐Apochromat ×63 (numerical aperture 1.4) lens. The DAPI, Oregon‐Green, and TMR fluorescence were detected at excitation wavelengths of 405, 488, and 555 nm with 300–483, 493–550, and 560–800 nm band‐pass filters, respectively. Images were processed by using zen 2012 imaging software (Carl Zeiss).

### Image analysis

KikGR‐Myh3 or Halo‐Myh3 images were line‐scanned and analyzed with imagej, which calculated an average line‐scanned value from the line‐scanned region of the sarcomere. Image data were applied for curve‐fitting with a polynomial to extract one peak corresponding to one sarcomere. Two turning points of the green fluorescence curve were defined as the Z‐bands. The red fluorescence curve was also extracted at the exact position of green fluorescence. The baseline was defined as a line between two turning points. The fluorescence intensity of green and red was normalized as a max value of 1. The horizontal axis (x‐axis) indicates that the normalized distance from the center of the sarcomere (M‐line) is 0.0, whereas the Z‐bands are located at 1.0 and −1.0.

For image analysis of KikGR‐Myh3, the average fluorescence intensities were calculated from unprocessed fluorescence intensities. Average waveforms at each time point were curve fitted with a polynomial. In normalized line‐scanned data, green fluorescence intensities were subtracted from the red fluorescence intensities to compare the waveforms of green and red fluorescence.

For image analysis of the fluorescence pulse‐chase assay using Halo‐Myh3, we compared the red waveform containing a peak(s) with the green waveform during a pulse‐chase assay and categorized them into eight patterns based on the red peak position: both sides, predominantly right side, predominantly left side, right side, left side, center, random fashion, and no incorporation. The red fluorescence peak shift was evaluated as the distance between the red fluorescence intensity peak and the center of the thick filament using graphs categorized into five patterns: both sides, predominantly right side, predominantly left side, right side, and left side. Waveform analysis and graph drawing were conducted with a home‐built program written in Colaboratory (Google Research, Tokyo, Japan).

### Statistics

All data are expressed as the mean ± standard error (SE). Student’s *t*‐test was used to compare differences between two groups. The Tukey test was used for multiple comparisons. Statistical significance was set at *P* < 0.05. All statistical tests were performed using EZR on R commander ver. 1.54.

## Results

### Simultaneous myosin incorporation and release were observed in the myofibrils

**Fig. 1:**
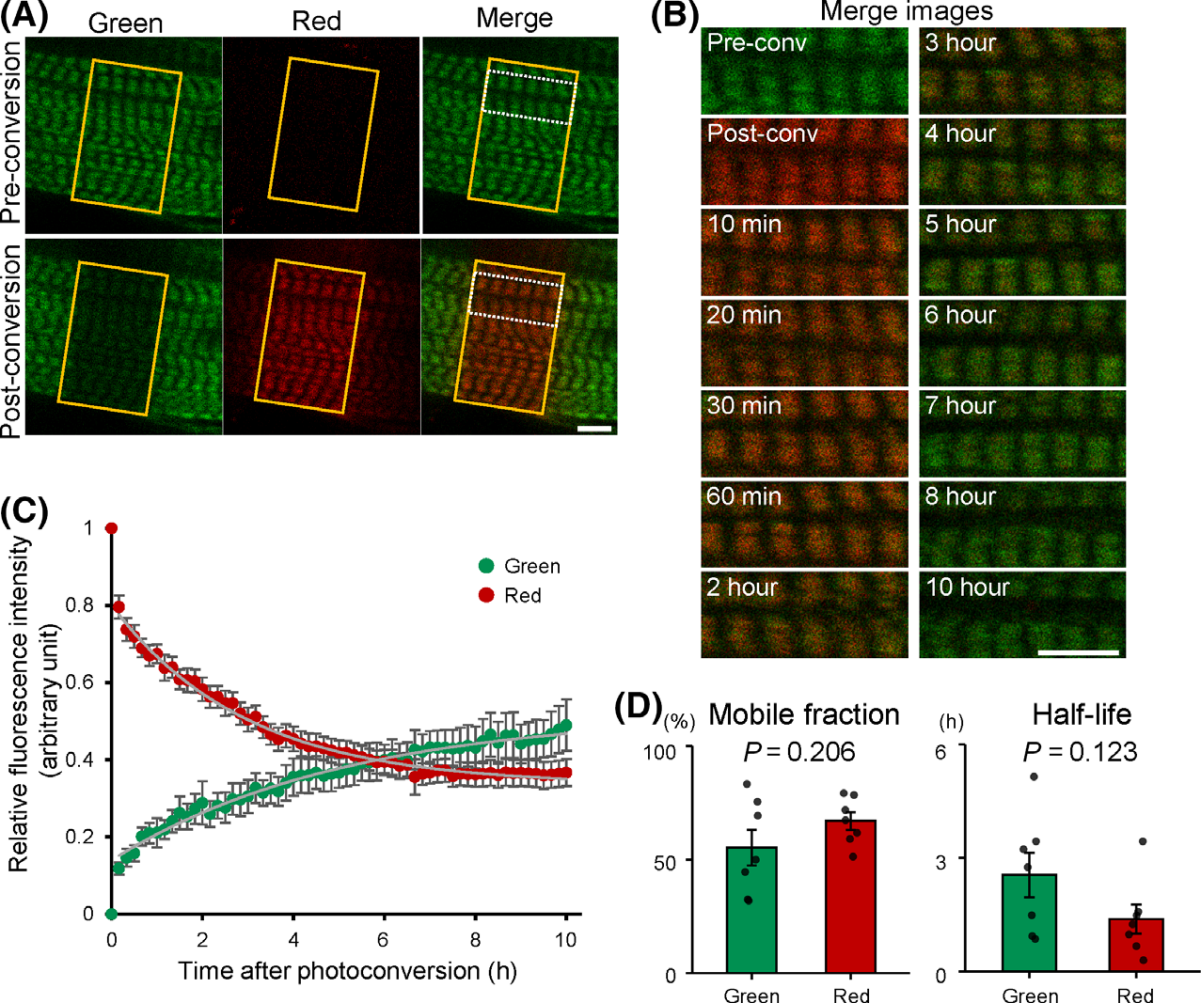
Photoconversion analysis of KikGR‐Myh3 in the myofibrils. (A‐B) Localization of photoconvertible KikGR‐Myh3 in the myofibrils. (A) A green fluorescence signal of KikGR‐Myh3 was detected in the thick filament of myofibrils at preconversion. The fluorescence color of KIkGR‐Myh3 shifted from green to red with exposure to UV light (yellow rectangles). Bar, 5 µm. (B) Alternation of KikGR‐Myh3 fluorescence signals was monitored at 10 min intervals up to 10 h after photoconversion. Merged images with green and red fluorescence signals are indicated in the dashed rectangular area in A. Bar, 5 µm. (C‐D) Symmetrical and biphasic changes of KikGR‐Myh3 fluorescence intensities in the myofibrils. (C) Green and red fluorescence signals of KikGR‐Myh3 were measured in the myofibrils at the indicated time points. The circle symbol of each color shows measurement value, and white curves show curve‐fitting results. (D) Mobile fractions (%) were 55.24 ± 7.86% for green fluorescence and 66.98 ± 3.90% for red fluorescence. T_1/2_ (h) were 2.55 ± 0.59 for green fluorescence and 1.38 ± 0.38 for red fluorescence. Values represent the mean ± SE. p values were calculated by Student’s *t‐test*. *n* = 7 for each group.

Our previous study demonstrated that thick filament‐associated myosin is briskly released with an exchange half‐life of ~ 3 h [19]. To understand how fast myosin is replaced in the thick filament, we measured the myosin replacement rate in cultured myotubes expressing a photoconvertible fluorescence protein, Kikume Green‐Red fused Myh3 (KikGR‐Myh3), whose fluorescence color irreversibly shifts from green to red with exposure to UV light [20]. The green fluorescence signal of KikGR‐Myh3 was observed in the myofibrils, whereas red fluorescence was not detected at preconversion (Fig. 1A). Upon exposure of the myotubes to UV light, green fluorescence was photoconverted to red fluorescence (postconversion in Fig. 1A). We were able to then clarify the dynamics of myosin incorporation and release, since the reduction of red fluorescence intensity and the increment of green fluorescence intensity reflect the myosin release and myosin incorporation, respectively. The identical focal plane of the myofibrils was selected to monitor green and red fluorescence signal intensities at 10 min intervals (Fig. 1B). Green fluorescence increased to ~ 10% of signal intensity in 10 min and ~ 20% in 60 min, while red fluorescence decreased by ~ 20% of signal intensity in 10 min and by ~ 30% in 60 min (Fig. 1C). The fluorescence intensities of green and red were symmetrically changed in 10 h. The fluorescence intensity of each color also showed a biphasic alteration, that is, the fluorescence intensity was changed drastically at the early phase of the FRAP experiment, then changed more gradually, and finally reached a plateau. Consequently, some myosin in the myofibrils was replaced within 10 min. Importantly, Mf and t_1/2_ were not significantly different between red and green fluorescence (Fig. 1D).

### Red fluorescence of KikGR‐Myh3 was released faster at the tip of the thick filament

**Fig. 2:**
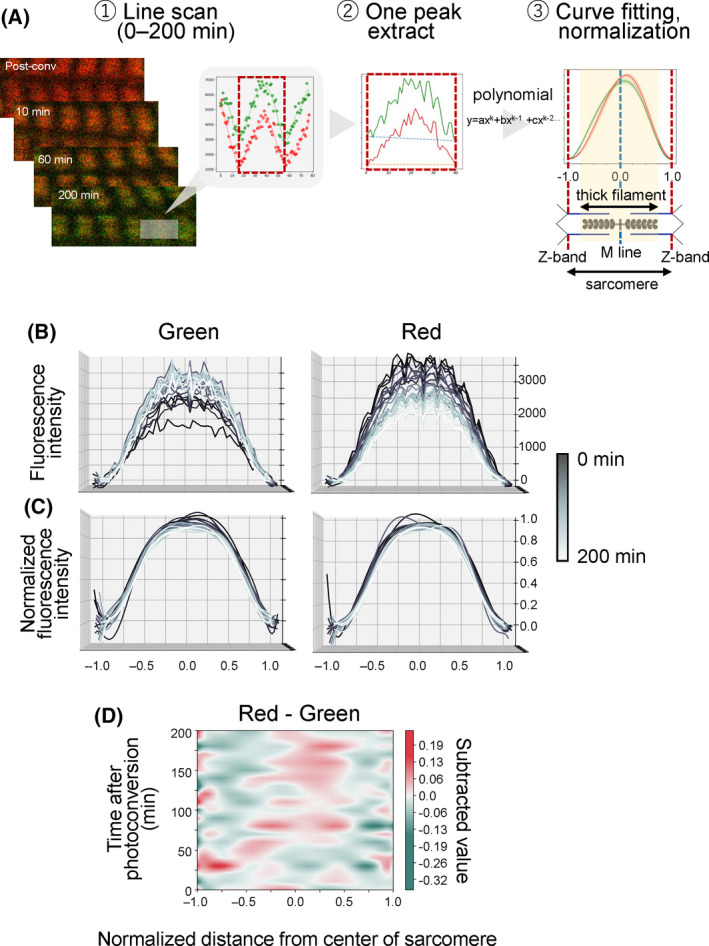
Changes across time in the green and red fluorescence distribution of KikGR‐Myh3 in the sarcomeres. (A) A scheme of image‐processing to compare the distributions of red and green fluorescence of KikGR‐Myh3 in each sarcomere. First, the fluorescence signal was line‐scanned along myofibrils to obtain mean fluorescence intensities in a sarcomere with 10 min intervals up to 200 min (dashed rectangular area in Fig. 1A). Second, a single peak between turning points of green fluorescence intensity was extracted as a fluorescence waveform. Finally, the data was curve fitted with a polynomial formula, and a fluorescence max value at the y‐axis and a max distance from the center at the x‐axis were normalized to 1.0. The normalized distance of −1.0~1.0 (between Z‐bands) indicates the width of a sarcomere, and 0.0 indicates the center of the sarcomere (M line). The two ends of the thick filament correspond to approximately −0.8 and 0.8. (B‐C) Overlaid graphs of fluorescence waveforms. Twenty line‐scanned images taken from 0 to 200 min at 10 min intervals following photoconversion were overlaid. The unprocessed fluorescence intensity and the normalized fluorescence intensity are shown in B and C, respectively. The x‐axis was the normalized distance from the center of the sarcomere. The y‐axis was the unprocessed (B) and the normalized (C) fluorescence intensities. *n* = 7. (D) The difference between the normalized green fluorescence intensity and normalized red fluorescence intensity. The subtraction images of green from red were drawn. The color code reflects the subtracted values of the normalized green signal intensity from the normalized red signal intensity. The x‐axis and the y‐axis indicate the normalized distance from the center of the sarcomere and the time post‐photoconversion (min). *n* = 7.

Our results showed that myosin release and insertion occur concurrently in the myofibrils, based on our measurement of the ratio of fluorescence signals in selected myofibrils. The next question was which regions within the thick filament were favored for myosin exchange. To answer this, we analyzed the patterns of myosin release and insertion at the thick filament level. The green and red fluorescence signals of each sarcomere were line‐scanned to draw fluorescence waveforms during the photoconversion experiments (Fig. 2A–C). Normalized green fluorescence signals were subtracted from normalized red fluorescence signals to calculate the difference of two fluorescence waveforms on the thick filaments during photoconversion experiments (Fig. 2D). We found that the red fluorescence intensity was greater than the green fluorescence intensity in the middle part of the thick filament after 60 min post‐photoconversion (Fig. 2D). By contrast, green fluorescence signal intensities were dominant in the region indicated by around −0.75 and 0.75 at the X‐axis in Fig. 2D, corresponding to the tip of the thick filaments.

### Newly synthesized myosin tends to be incorporated into the tip of the thick filament

**Fig. 3:**
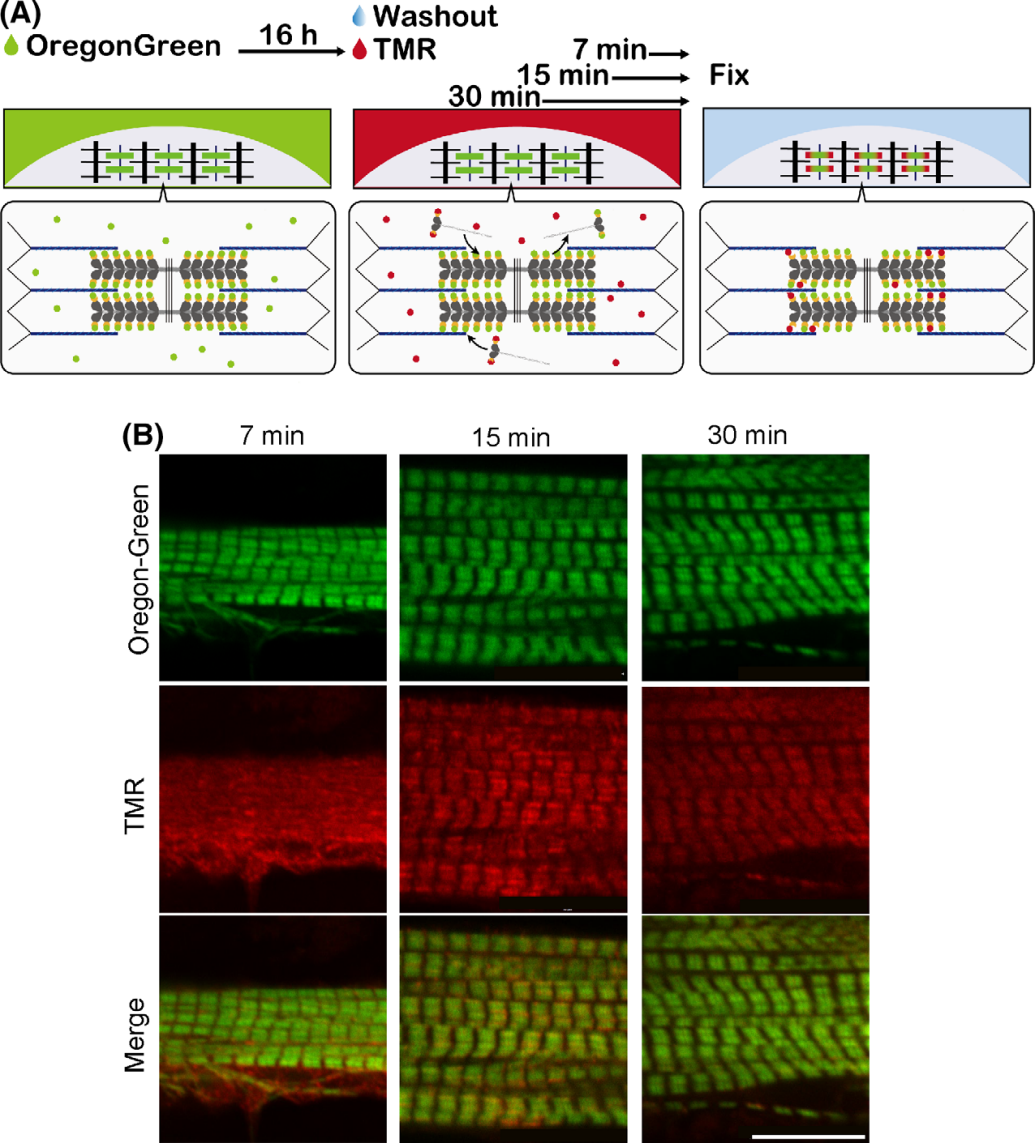
Visualization of the incorporation of newly synthesized myosin into the thick filaments. (A) Experimental procedure of different fluorescence pulse‐chase labeling using Halo‐Myh3. First, myotubes expressing Halo‐Myh3 were incubated with the Oregon‐Green ligand (green fluorescence) for 16 h. Next, myotubes were reacted with the TMR ligand (red fluorescence) to label newly synthesized myosin for 7, 15, or 30 min after washout of Oregon‐Green ligand. Finally, myotubes were fixed and observed by confocal microscopy. (B) Representative images of myotubes expressing Halo‐Myh3 with fluorescence pulse‐chase labeling. Bar, 10 µm.

Our KikGR‐Myh3 experiments suggest a trend in which the thick filament‐associated myosin is released more frequently from the tip of the thick filaments than the middle of the thick filaments. We took advantage of a fluorescence pulse‐chase assay technique to determine the myosin‐specific incorporation region in the thick filament of myotubes expressing Halo‐Myh3, which irreversibly binds to the fluorescence‐conjugated cell membrane permeable ligand. The Halo‐tag technique enabled us to track newly synthesized Halo‐Myh3 with different fluorescence ligands. First, expressed Halo‐Myh3 was labeled with the Oregon‐Green (green fluorescence) ligand for 16 h, and then, Halo‐Myh3 was labeled with the TMR (red fluorescence) ligand for 7, 15, or 30 min (Fig. 3A). Green fluorescence‐labeled Halo‐Myh3 was observed in the thick filaments during the pulse‐chase experiment. Following the 7 min incubation, red fluorescence‐labeled Halo‐Myh3 was localized to the myofibrils with diffuse cytoplasm (Fig. 3B). After the 15 and 30 min incubations, red fluorescence‐labeled thick filaments were more prominently visualized.

**Fig. 4:**
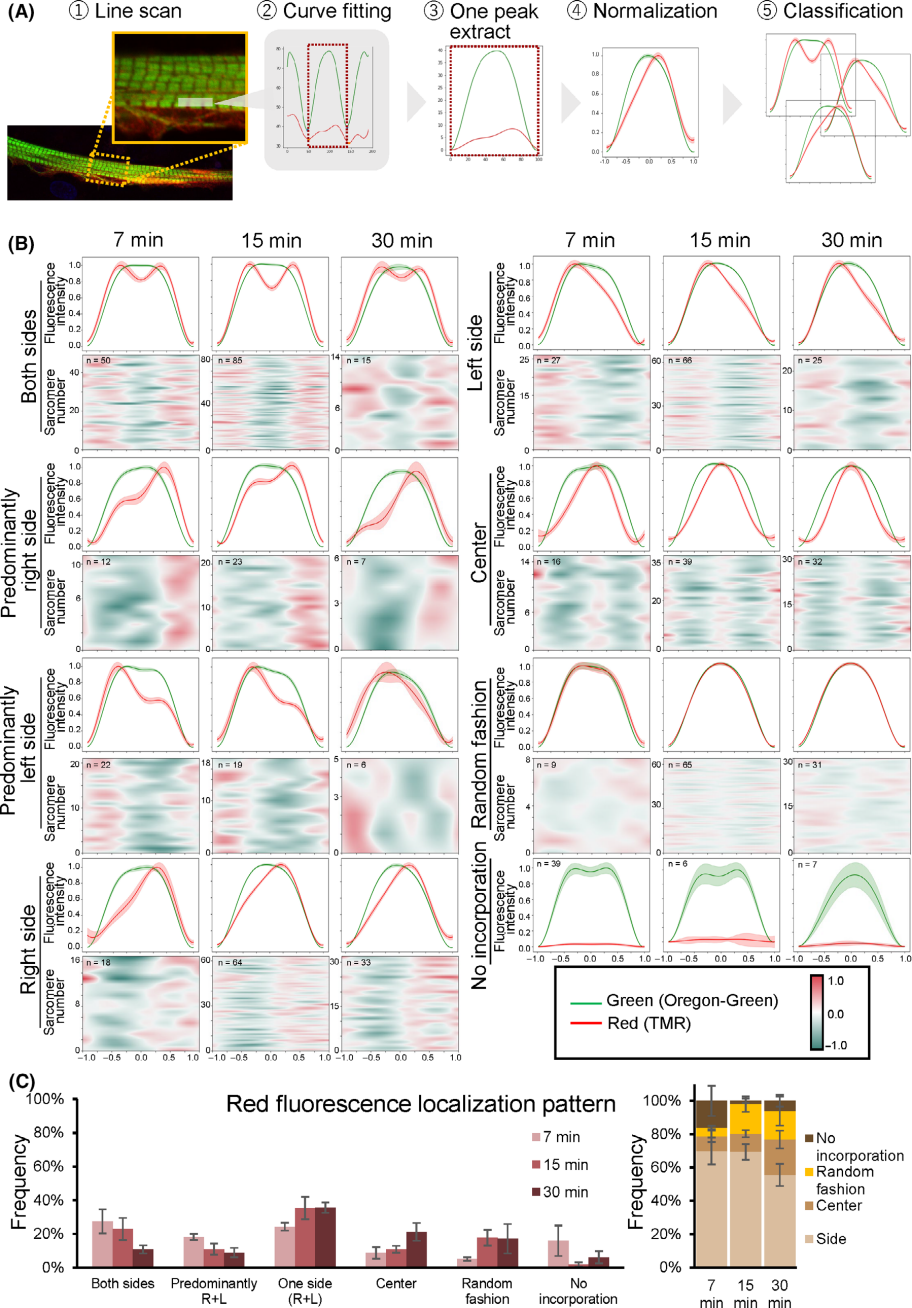
A variety of incorporation patterns of newly synthesized Halo‐Myh3 into the thick filaments. (A) A scheme to categorize newly synthesized myosin insertion sites in the thick filaments. First, each sarcomere was line‐scanned. Second, line‐scanned data were curve‐fitted with a polynomial formula. Third, a green waveform and its corresponding red waveform were extracted. Fourth, the green and red fluorescence intensities were normalized as a max value of 1. Finally, waveforms were classified into eight patterns based on the red peak position. (B) Eight patterns of newly synthesized Halo‐Myh3 incorporation into the thick filaments. Line‐scanned images were categorized into eight groups based on the waveforms of red fluorescence signals compared to green fluorescence signals: both sides, predominantly right side, predominantly left side, right side, left side, center, random fashion, and no incorporation. Solid lines show the average value ± SE (pale‐colored width) in the upper graphs. The lower panels are drawn by enumeration of individual subtraction of the green fluorescence signal from the red fluorescence signal. In the no incorporation group, red fluorescence intensities are shown as the relative values against green fluorescence intensities. The x‐axis indicates the normalized distance from the center of the sarcomere indicated as 0.0. The normalized distance between the center of the sarcomere and the Z‐bands was indicated as 1.0 and −1.0. The y‐axis indicates the normalized fluorescence intensity in the upper panels and the sarcomere number examined in the lower panels. (C) Frequency pattern of myosin insertion into the thick filament. The bar chart indicates the ratio of the myosin incorporation pattern. The stacked bar graph shows the proportion of side groups (both sides and either side), center, random fashion, and no incorporation. Values represent the mean ± SE. 7 min, *n* = 193 in 3 myotubes. 15 min, *n* = 367 in 9 myotubes. 30 min, *n* = 156 in 6 myotubes.

Next, each sarcomere was line‐scanned to determine the region of incorporation of the newly synthesized red fluorescence‐labeled myosin into the thick filament and the fluorescence patterns were classified (Fig. 4A). We categorized red fluorescence patterns into eight groups based on the regions of the red peak signal in the thick filaments: both sides, predominantly right side, predominantly left side, right side, left side, center, random fashion, and no incorporation (Fig. 4B). Average values in each category are shown in the upper graphs in Fig. 4B. Enumeration of the individual data in the lower panels of Fig. 4B showed that the normalized green fluorescence intensities were subtracted from the normalized red fluorescence intensities. The most frequent localization patterns were those involving the side groups, that is, the both sides, left side and right side patterns during pulse‐chase (~ 80% in each time point: Fig. 4C). The frequency of the center pattern gradually increased to ~ 20% at 30 min of incubation. The random pattern, in which the red and green fluorescence signal patterns were identical, slightly increased to ~ 20% at 15 min. The frequency of the no signal pattern, defined as no red fluorescence, was decreased in 15 min on incubation (Fig. 4C).

**Fig. 5:**
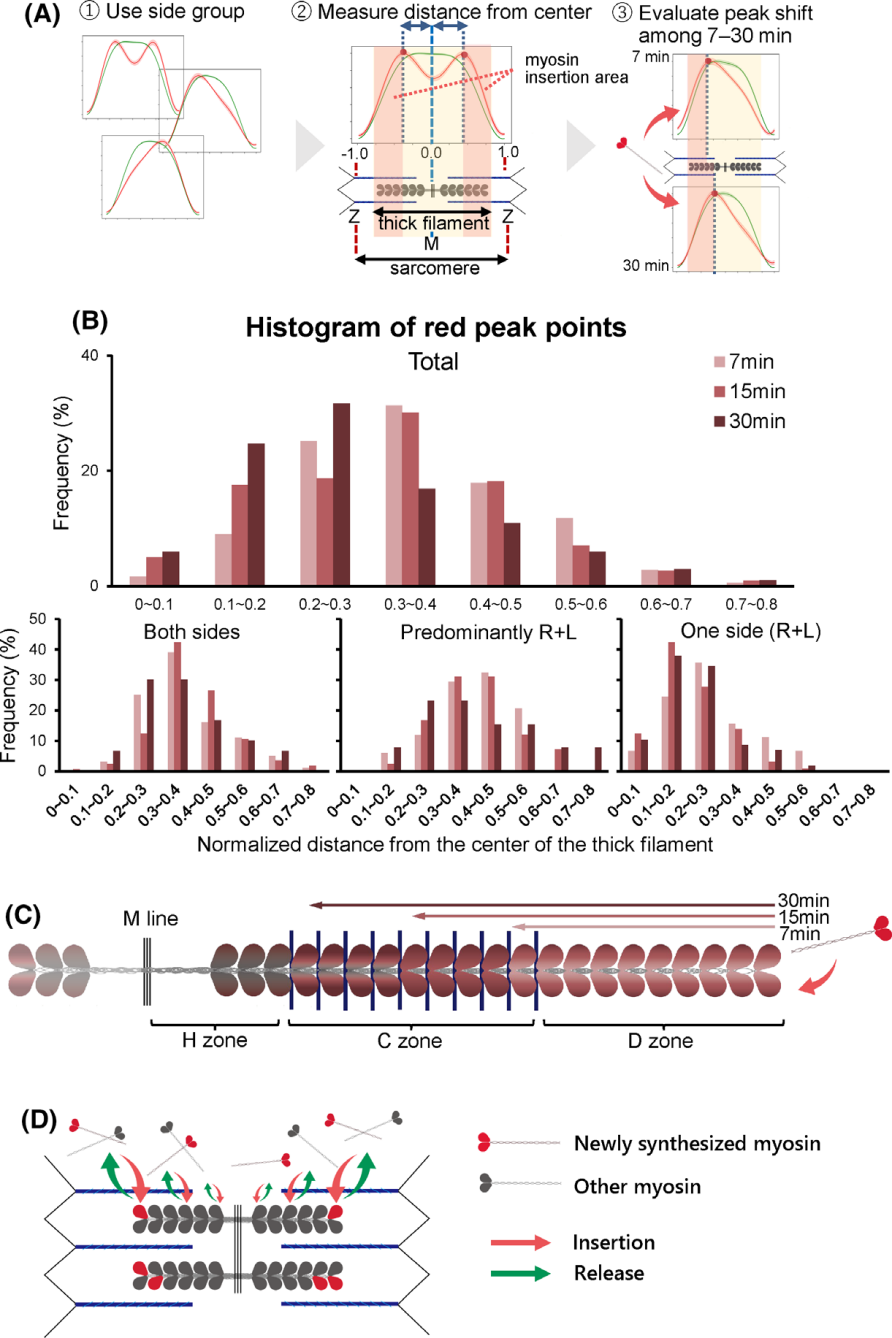
Comparison of distance between the thick filament center and the peak signal of Halo‐Myh3 with red fluorescence. (A) A scheme to evaluate the insertion site of newly synthesized myosin with red fluorescence on the thick filaments. First, the insertion area of newly synthesized myosin was compared using waveforms categorized to the side group. Second, the distance between the red fluorescence peak and center was measured as the myosin insertion area from the tips of the thick filament to red fluorescence peak point(s). Finally, the measured distance was plotted in the histogram and the peak shift was evaluated during pulse‐chasing labeling. (B) A histogram of newly synthesized myosin incorporation sites on the thick filaments. The frequency of the myosin incorporation site, defined as the distance between the red fluorescence peak and the center of the thick filament, is shown (upper panel). The frequency was further divided into the three categories: Both sides, Predominantly R + L, and One side (R + L) (lower panel). The x‐axis indicates the normalized distance from the center of the thick filament. The center of the thick filament is located at 0, and the tip of the thick filament is located at approximately 0.7–0.8. The numbers of thick filaments at each time point were as follows: 7 min, *n* = 129 in 3 myotubes; 15 min, *n* = 257 in 9 myotubes; 30 min, *n* = 86 in 6 myotubes. (C) Transition of newly synthesized myosin insertion into the thick filaments. The shift of the red fluorescence peak from the tip to the center of the thick filament indicates that the red fluorescence‐tagged myosin was inserted into the region closer to the center of the thick filament over time. The color of the thick filament reflects the frequency of myosin insertion, that is, increasing saturation of the red color means higher frequency. (D) A model of myosin replacement in the thick filament. When thick filament‐associated myosin is replaced, (1) the individual myosin molecule acts as an exchange unit, (2) myosin release and insertion occur concurrently, and (3) myosin is more frequently replaced at the tip of the thick filament. The size of the arrow reflects the myosin replacement rate in the thick filament conceptually.

Finally, we investigated where newly synthesized myosin was inserted into the thick filament. For this purpose, we plotted the distance between the red fluorescence peak and the center of the thick filament during pulse‐chase labeling using the data for the side groups, since the localization patterns involving the side groups were the most frequent (Fig. 5A). The distance between the red fluorescence peak and the center of the thick filament was lessened during pulse‐chase labeling (Fig. 5B). The myosin insertion area in the thick filaments was gradually spread from the tip of the thick filaments towards the center of the thick filaments as the red fluorescence peak shifted to the center of the thick filaments. These results indicate that newly synthesized myosin is not evenly replaced in the thick filament, that is, newly synthesized myosin tends to be incorporated into the tips of the thick filament (Fig. 5C). Our findings showed that the replacement rate of the thick filament‐associated myosin varies depending on the regions on the thick filament, with the myosin replacement rate being faster at the tip than the center of the thick filament (Fig. 5D).

## Discussion

In this study, we demonstrated the dynamic exchange of myosin in the thick filament of cultured myotubes by means of a live imaging technique. Our results showed that (a) myosin itself is the unit of replacement, (b) not all, but some myosins can be rapidly replaced by other myosins within 7–10 min in the thick filaments of myotubes; and (c) the myosin replacement frequency differs depending on the region of the thick filament. Based on our observations, we propose the model shown in Fig. 5D to summarize the dynamics of myosin replacement in the thick filament.

We found that the thick filament‐associated myosin had the capacity to be promptly replaced in min. Although our previous studies showed that about 50% of eGFP‐Myh3 is replaced in the myofibrils in ~ 3 h, in those studies we did not investigate the myosin replacement rate over a brief period of time [19]. The present KikGR‐Myh3 experiments revealed that approximately 10% of myosin in the thick filament was replaced in only 10 min (Fig. 1). Our fluorescence pulse‐chase assay also showed that incorporation of myosin into the thick filament was observed within 10 min (Fig. 3 and 4). This quick myosin exchange in the thick filament may in part reflect the biochemical properties of myosin, since synthetic myosin filaments are extensively exchanged for other myosin in minutes *in vitro* [21, 22, 23]. Although the thick filament‐associated myosin molecules are rapidly and continuously replaced by newly synthesized myosin and the cytosolic myosin, it takes ~ 3 h to exchange half of the myosin molecules in the thick filament (Fig. 1, [19]); this relatively protracted amount of time is attributable to the complexity of the thick filament, which contains about 300 myosins with the thick filament‐associated proteins (see below).

Our KikGR‐Myh3 experiments showed that the thick filament‐associated myosin release and the myosin incorporation into the thick filament occur concurrently. We found different myosin replacement rates along the thick filament. Moreover, the thick filament‐associated proteins, such as MybpC and myomesin, are also exchanged by others in the thick filaments, but their replacement rates are independent of myosin [19]. These results support the notion that the replacement unit in the myofibril is a myosin molecule rather than a thick filament.

Based on the following observations, we conclude that myosin is the most frequently replaced at the tip of the thick filament (Fig. 5D). First, the red fluorescence intensity of KikGR‐Myh3 was rapidly reduced at the tips of the thick filaments compared to the middle of the thick filaments. Second, newly synthesized myosin tended to be inserted into the tips of the thick filaments. Third, both the red and green intensities of KikGR‐Myh3 changed biphasically. Our results are consistent with the following results from several pioneering works. An *in vitro* biochemical study showed that myosin was exchanged at the tips of the synthetic myosin filament [23]. In glycerinated myofibrils, the thick filament dissociated from both ends under high ionic strength conditions [24]. Finally, a study using an immune‐electron microscopic technique revealed a greater exchange of new myosin at the ends of the thick filament than at other regions in rabbit cardiac cells [25]. Therefore, the tip is the region with the briskest exchange of myosin in the thick filament of skeletal muscle cells as well as in the filaments of cardiomyocytes and *in vitro* synthetic myosin.

Our results showed the different myosin replacement rates on the thick filament. The complexity of the thick filament may partly explain these results. The thick filament is a bipolar structure that is separated into several parts [26]. The center of the thick filament is called the H zone, where myosin is arranged in an antiparallel fashion. A bundle of thick filaments is tied with myomesin in the H zone. Seven to eleven MybpC striped with about 43 nm intervals on the thick filament are called the C zone [5, 26, 27]. Both ends of the thick filament are called the D zone [28]. In addition, connectin/titin also interacts with the thick filament at multiple sites in the H and the C zones [5, 10]. Unlike the thin filament, whose ends are capped with capping proteins such as tropomodulin at the pointed end and Cap‐Z at the barbed end [29, 30], both ends of the thick filament are uncapped, leading to frequent myosin exchange at both ends of the thick filaments. These structural components of the thick filaments might be obstacles to the more prompt exchange (release and insertion) of myosin on the H and the C zones of the thick filaments compared to the D zones. Furthermore, the fact that the difference in the frequency of myosin replacement is dependent on the position within the thick filament might be the reason for the biphasic replacement of myosin observed in our KikGR‐Myh3 experiments.

Collectively, our results show that myosin is briskly and continuously replaced in the thick filaments in living myotubes. As *in vitro* biochemical studies demonstrated that myosin filaments are in equilibrium with a free myosin pools [21, 22, 23], the dynamic equilibrium of myosin is also involved in the myosin replacement in myotubes. The frequent exchange of thick filament‐associated myosin may play a role in hypertrophy and atrophy of myofibers as well as in myosin isoform shift from embryo to neonate or adult during muscle development. In this context, myosin might be selectively replaced by other myosin isoforms rather than stochastically replaced under particular conditions as in case of myosin replacement facilitated by myosin ubiquitin ligase selectively breaking down a specific myosin isoform [31, 32].

## Conflict of interest

The authors have no conflict of interest to declare.

## Data accessibility

The data that support the findings of this study are available from the corresponding author (nishi@anim.agr.hokudai.ac.jp) upon reasonable request.

## Author contributions

EI, KO, and TN designed the research; EI and KO performed the experiments; EI, KO, SM, and KK analyzed the data; EI, KO, and TN wrote the manuscript.
